# Soil bacterial communities associated with marbled fruit in *Citrus reticulata* Blanco ‘Orah’

**DOI:** 10.3389/fpls.2023.1098042

**Published:** 2023-05-08

**Authors:** Qichun Huang, Nina Wang, Jimin Liu, Huihong Liao, Zhikang Zeng, Chengxiao Hu, Chizhang Wei, Songyue Tan, Fuping Liu, Guoguo Li, Hongming Huang, Dongkui Chen, Shaolong Wei, Zelin Qin

**Affiliations:** ^1^ Horticulture Research Institute, Guangxi Academy of Agricultural Sciences, Nanning, China; ^2^ College of Resources and Environment, Huazhong Agricultural University, Wuhan, China; ^3^ Institute of Plant Protection, Guangxi Academy of Agricultural Sciences, Nanning, China; ^4^ College of Plant Science and Technology, Huazhong Agricultural University, Wuhan, China; ^5^ Institute of Agricultural Science and Technology Information, Guangxi Academy of Agricultural Sciences, Nanning, China; ^6^ Guangxi Academy of Agricultural Sciences, Nanning, China

**Keywords:** orah, marbled fruit, 16S rRNA sequencing, soil bacterial communities, pathways

## Abstract

*Citrus reticulata* Blanco ‘Orah’ is grown throughout southern China and provides enormous economic value. However, the agricultural industry has suffered substantial losses during recent years due to marbled fruit disease. The present study focuses on the soil bacterial communities associated with marbled fruit in ‘Orah’. The agronomic traits and microbiomes of plants with normal and marbled fruit from three different orchards were compared. No significant differences were found in agronomic traits between the groups, except for higher fruit yields and higher quality of fruits in normal fruit group. Additionally, a total of 2,106,050 16S rRNA gene sequences were generated *via* the NovoSeq 6000. The alpha diversity index (including the Shannon and Simpson indices), Bray–Curtis similarity, and principal component analyses indicated no significant differences in microbiome diversity between normal and marbled fruit groups. For the healthy ‘Orah’, the most abundant associated phyla were Bacteroidetes, Firmicutes, and Proteobacteria. In comparison, Burkholderiaceae and Acidobacteria were the most abundant taxa with the marbled fruit group. In addition, the family Xanthomonadaceae and the genus *Candidatus Nitrosotalea* were prevalent with this group. Analysis using the Kyoto Encyclopedia of Genes and Genomes pathways showed that several pathways related to metabolism significantly differed between the groups. Thus, the present study provides valuable information regarding soil bacterial communities associated with marbled fruit in ‘Orah’.

## 1 Introduction

Soil health is a vital factor for plants and provides for a central living ecosystem and crop yields (Lehmann et al., 2020). Soil health can be affected by climate, mineral composition, organic content, and biotic factors (Turmel et al., 2015; Lal, 2016). Recent studies have shown that soil microbiota influence plant growth and yields (Bulgarelli et al., 2013; Khodakovskaya et al., 2013; Tkacz and Poole, 2015). Microbiota are complex systems of microbial communities that provide important proteins, such as enzymatic resources, for plant roots (Gianfreda and Rao, 2008). The rapid development of next-generation high-throughput sequencing and bioinformatic technologies has revealed new information about the function of microbiota. The connections between plants and the soil microbiota around their roots are critical for nutrient absorption, metabolism, and growth (Hacquard et al., 2015; Chialva et al., 2022).

*Citrus reticulata* Blanco ‘Orah’ is a small, fruiting citrus tree that was bred by Spiegel-Roy and Vardi (Usman and Fatima, 2018; Qin et al., 2022). To date, this variety has been planted throughout southern China and has produced substantial income for farmers and high-quality fruits for consumers (He et al., 2022). Although ‘Orah’ orchards have been expanding rapidly in recent years, the plants suffer from several diseases, such as marbled fruit (Liu et al., 2020b). Marbled fruit occurs in several citrus varieties and results in shrunken and light-weight fruit. Boron deficiency and citrus yellow vein clearing virus can also lead to marbled fruit (Liu et al., 2019). Furthermore, grafting, scions, seedlings, and indirect contact by tools can spread the disease, causing large financial losses. Therefore, understanding the pathogeny of this disease would improve the production of ‘Orah’.

Identification of soil-based probiotics provides potential therapies for plant diseases (Wu et al., 2020). For example, irrigation of plant roots with *Bacillus subtilis* has been found to alleviate wilt disease in watermelon (*Citrullus lanatus*) (Ge et al., 2021). *Bacillus amyloliquefaciens* inhibits pathogenic bacteria, including *Gymnosporangium asiaticum*, *Phytophtora parasitica*, and *Pythium helicoides* (Asari et al., 2016; Ngalimat et al., 2021). Furthermore, investigation of soil microbiomes from the roots of diseased plants may identify possible pathogens, thereby aiding in providing precise and specific therapies for marbled fruit disease. Unfortunately, data on the microbiome remains scarce, resulting in a lack of key evidence regarding the pathogeny of marbled fruit disease in ‘Orah’.

Nevertheless, this is among the most concerning diseases in ‘Orah’. The current treatment strategies include increasing nutrition by fertilizing, replenishing the beneficial microbiome, sod-culture, and hormonal control (Qin et al., 2022). These therapies provide clear effects against marbled fruit disease; however, they still have several defects. First, these therapies are concerned with the whole environment of the plants rather than the inhibition of a specific pathogen. Additionally, these therapies are complicated to use. Thus, more details are required regarding the pathogenesis of marbled fruit disease in ‘Orah’. In the present study, we compared normal, healthy fruits (NF) and marbled fruits (MF) from three different orchards. The plant and fruit morphologies of the NF and MF groups were examined. In addition, the soil microbiomes from the roots of these groups were evaluated *via* 16S rRNA gene sequencing using a next-generation approach. Finally, we analyzed the differences in diversity, taxa, and functions of the microbiome between NF and MF. This information will will provide potential information of marble fruit disease for ‘Orah’.

## 2 Materials and methods

### 2.1 Field experiments and sampling

The tested ‘Orah’ trees were growing in three different orchards in Wuming, Naning, China, specifically Guangxi Jiacai Ecological Agriculture Co., Ltd (Wuming, Nanning, China; named JC), Guangxi Nanning Wanjin Agriculture Co., Ltd (Wuming, Nanning, China; named WJ), and Xiaoleima village (Wuming, Nanning, China; named XLM). The plants were sampled from 2019–2021. All the trees were four years old and randomly selected in the present study. The plants were managed following standardized fertilization and management techniques as described by (Huang et al., 2022). All the samples and tested groups was blind to the investigators in the study. Twenty individuals from each group were included in the study. The soil samples were collected from the root of the tree in 20 cm depth. For each group, 100 g soil samples from 5 trees were collected and 3 replications were performed. We used 0.5 g soil from each sample for DNA extraction. For analysis of agronomic traits, 10 fruits from each tree were used for the present study. The soil samples were talking about the soil around the plant roots.

### 2.2 Measurements of plant and fruit quality

The plant height, stem diameter, leaf thickness, leaf length, leaf width, number of fruits, and percentage of NF were calculated following the methods of (He et al., 2022). Leaf chlorophyll content was determined as described by (Zhu et al., 2020) using a SPAD-502 chlorophyll meter (Konica Minolta Inc., Japan).

The histology of leaves and fruit peel from the NF and MF groups were observed *via* paraffin section as described by (Zhang et al., 2021). Briefly, the leaf and fruit peel tissues were cut into 2–3 mm sections and embedded in paraffin. The tissues were then cut into 5 μm slices. Subsequently, the slices were dewaxed using xylene and the tissues were stained using safranine and fast-green followed by neutral gum sealing. Finally, the sections were observed and photographed using a DM2500 optical microscope (Leica Microsystems, USA).

### 2.3 DNA extraction and sequencing

DNA was extracted from the samples using a TIANamp Soil DNA Kit (TIANGEN, China) according to the manufacturer’s protocols. The quality of the DNA was measured using a 1% agarose gel and NanoDrop 2000 spectrophotometer (NanoDrop, USA). The 16S rRNA genes from partial bacterial DNA fragments were amplified *via* touchdown polymerase chain reaction (PCR). The primers for amplification of the variable regions, which included V3-V4 of the 16S rRNA genes, were 341F: 5′-CCTAYGGGRBGCASCAG and 806R: 5′-GGACTACNNGGGTATCTAAT (Chialva et al., 2022). Three biological replicates of the groups were included in the study. For each sample, PCR was performed on three replicates in a reaction system with a total volume of 30 μL, composed of 15 μL of Phusion^®^ High-Fidelity PCR Master Mix (New England Biolabs), 2.5 μL of each primer (10 μM), sterile water, and 10 μL of DNA (1 ng/μL). The reaction was performed as follows: initial denaturation at 98°C for 30 s, followed by 25 cycles (98°C for 10 s, 55°C for 30 s, and 72°C for 30 s), and a final extension at 72°C for 5 min. The products were purified using agarose gel and Agencourt Ampure XP beads (Beckman, USA) according to the manufacturer’s instructions. The DNA samples were assayed using a PicoGreen dsDNA quantitation assay (Thermo Fisher, USA). Sequencing libraries were constructed using a TruSeq^®^ DNA PCR-Free Sample Preparation Kit (Cat number: FC-121-3003, Illumina, USA) following the manufacturer’s instructions, with the addition of index codes assayed using a Qubit@ 2.0 fluorometer (Cat number: Q33216, Thermo Fisher, USA) and Agilent Bioanalyzer 2100 (Cat number: 2100-1, Agilent, USA). Finally, the prepared DNA libraries were sequenced using an Illumina NovoSeq 6000 platform (Illumina, USA).

### 2.4 Bioinformatic analysis

The raw reads were first filtered to remove low-quality sequences, tags, and primers; subsequently, the non-bacterial ribosome sequences and chimeras were removed. The pair-end reads were assembled using FLASH v1.2.11 (Liu et al., 2021) and the assembled sequences were clustered into operational taxonomic units using the CD-HIT algorithm within the UCLUST program (USEARCH V11; https://www.drive5.com/usearch/). The alpha and beta diversity comparisons were performed using QIIME2 plugins. The similarity matrices were used Bray-Curtis distances and the s distances on square-root transformed abundance data were calculated using R packages “phyloseq”, “dplyr” and “ggplot2” (Yin et al., 2013). Functional analysis utilizing the Kyoto Encyclopedia of Genes and Genomes (KEGG) pathways was performed using MicrobiomeAnalyst (https://www.microbiomeanalyst.ca/MicrobiomeAnalyst/home.xhtml).

### 2.5 Statistical analysis

The statistical analysis and plotting were performed using R project (V4.2.1). The alpha and beta diversities were normalized prior to analysis using the read counts. The Shannon diversity index was used to reflect the alpha diversity. The Bray–Curtis dissimilarity matrix and permutational analysis of variance were used to assess the beta diversity which were analyzed by PRIMER v. 6 (PRIMER-E, UK). For the different groups, a principal component analysis (PCA) graph was plotted to show the clustering of the bacteria.

## 3 Results

### 3.1 Plant and fruit quality of ‘Orah’

**Figure 1:**
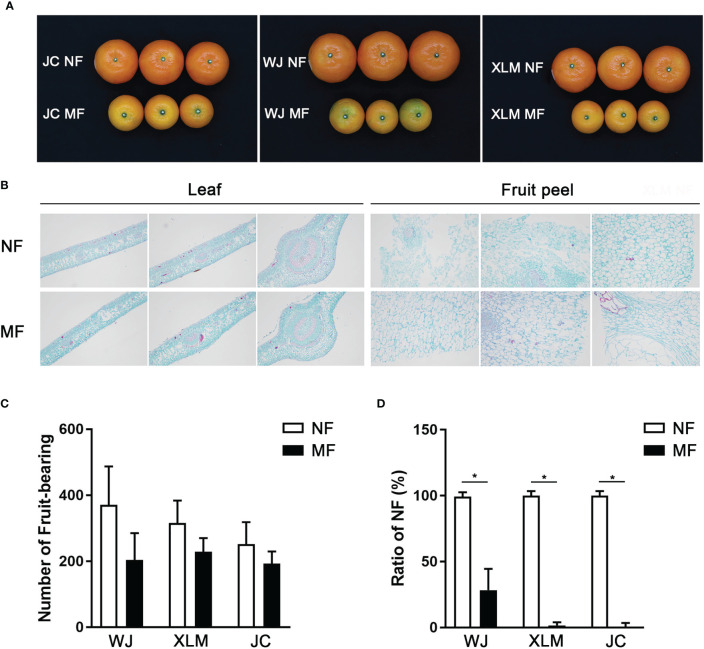
Comparisons of leaves and fruits between MF and NF groups. **(A)** The fruits in the MF and NF groups at the WJ, XLM, and JC orchards. **(B)** Histology of the leaves and fruit peel of MF and NF groups, which were analyzed *via* safranine and fast-green. **(C)** Statistical analysis of the number of fruits produced in the MF and NF groups at WJ, XLM, and JC. **(D)** Percentage of NF in the MF and NF groups at WJ, XLM, and JC. The asterisks show the significant differences between the two groups (P<0.05) by t-test.

First, the trees that produced normal and marbled fruit were compared (**Figure 1A**). The plant heights, tree trunk, leaf thicknesses, leaf lengths, leaf widths, and leaf chlorophyll contents showed no significant differences between the NF and MF from the three orchards (p>0.05, **Table 1**). The leaf histology did not differ between the NF and MF groups; however, the fruit peel contained more lignin in MF than that in NF (**Figure 1B**).

**Table 1:** comparison of the NF and MF plants from three planting areas.

Groups	Plant height (cm)	Tree trunk (mm)	Leaf thickness (mm)	Leaf length (mm)	Leaf width (mm)	SPAD	Number of fruiting-bearing	Ratio of NF (%)
WJ NF	272.67±6.96aA	76.82±3.65abAB	0.35±0.03abA	9.34±0.48aA	4.82±0.37aA	79.26±1.56aA	371.00±116.29aA	99.48±3.25aA
XLM NF	218.00±16.35bB	65.49±7.62cBC	0.33±0.01abA	7.85±0.05bB	4.35±0.09abA	75.68±0.54abA	316.00±68.13abA	100.00±3.53aA
JC NF	204.67±7.30bB	62.59±2.38cC	0.31±0.01bA	8.15±0.33bAB	4.46±0.18abA	73.33±4.63bA	252.33±66.54abA	100.00±3.53aA
WJ MF	282±22.37aA	81.71±5.75aA	0.37±0.03aA	8.47±0.82abAB	4.25±0.30bA	74.49±1.12abA	203.67±81.44bA	28.39±16.20bB
XLM MF	226.33±2.11bB	65.36±0.65cBC	0.33±0abA	8.19±0.45bAB	4.51±0.04abA	75.55±2.20abA	229.00±41.54abA	1.69±2.41cC
JCJ MF	203.67±8.48bB	70.60±3.69bcABC	0.33±0.03abA	8.01±0.38bAB	4.43±0.36abA	75.91±3.46abA	193.00±36.79bA	0±3.53cC

The lowercase letters and uppercase letters after the values showed significant differences at p < 0.05 and p < 0.01, respectively.

The MF contained more lignified cells than did the NF, which are shown stained red by Safranine in **Figure 1A**. However, the number of fruits produced did not significantly differ between the NF and MF groups (p>0.05, **Figure 1C**). The percentage of NF was significantly higher in the NF group than that in the MF group (p<0.05, **Figure 1D**).

### 3.2 Alpha and beta diversity analyses

The present study obtained a total of 2,106,050 16S rRNA gene sequences, including 1,754,017 sequences (83.28%) that represented a total of 7,889 effective operational taxonomic units (**Supplementary Table S1**), which were mostly assigned to 10 phyla (91.29–94.62%): Firmicutes, Chloroflexi, Proteobacteria, Bacteroidetes, Acidobacteria, Actinobacteria, Verrucomicrobia, Cyanobacteria, Thermotogae, and Thaumarchaeota (**Supplementary Table 2**).

**Figure 2:**
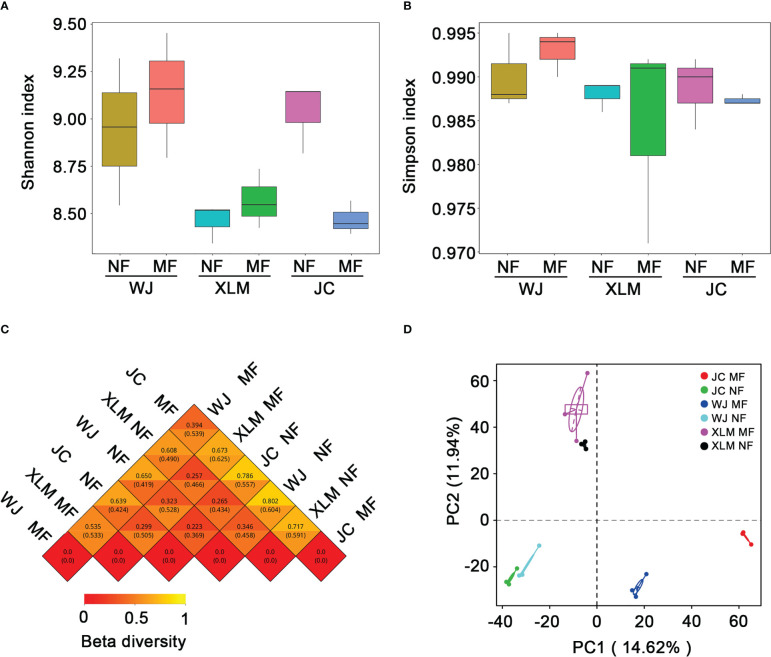
Alpha and beta diversity analyses of the samples from the MF and NF groups. **(A)** Shannon index and **(B)** Simpson index for alpha diversity of the samples in the MF and NF groups. **(C)** Correlations of beta diversity and **(D)** PCA among the groups in the present study.

We used the alpha diversity to calculate the Shannon and Simpson indices of the different genera. The Shannon index indicated no significant differences in the average diversity of the NF and MF groups at all orchards (p>0.05, **Figure 2A**). In addition, similar results were found when using the Simpson index to compare these groups (p>0.05, **Figure 2B**). To compare the overall divergence in the bacterial community compositions among the tested groups, the Bray-Curtis similarity and PCA were utilized (**Figures 2C, D**). Both analyses suggested that in the XLM, the NF and MF groups were highly similar to each other. However, in WJ and JC orchards, the NF and MF groups were most similar to the fruit same group from the other orchard.

### 3.3 Bacterial diversity in soil from roots of healthy ‘Orah’

**Figure 3:**
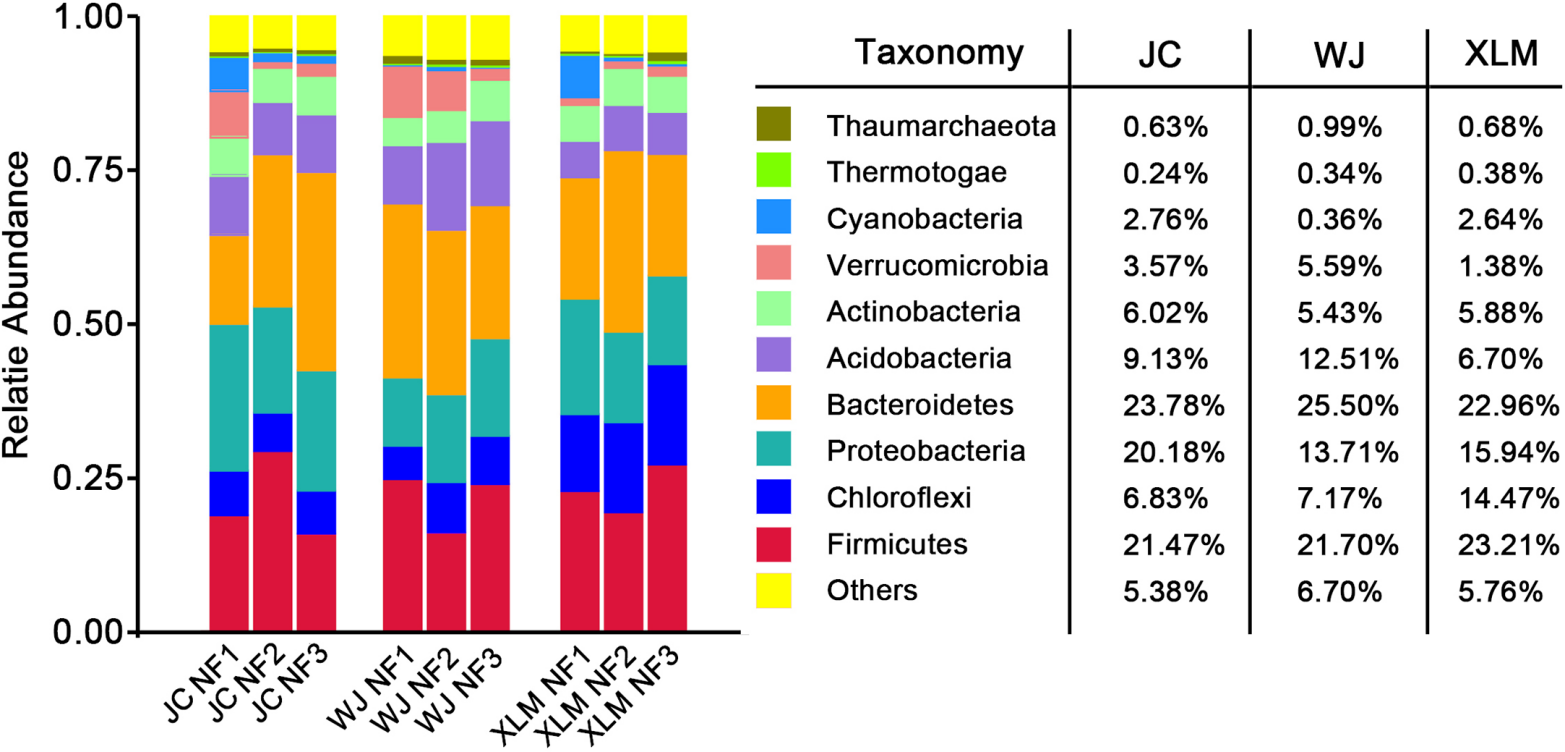
The 10 most abundant phyla of the NF group at JC, WJ and XLM.

The 10 most abundant phyla associated with the NF groups are shown in **Figure 3**. For the three orchards, the predominant bacterial phylum was Bacteroidetes (24.08%), followed by Firmicutes (22.13%) and Proteobacteria (16.61%). Moreover, the most abundant bacterial phylum at XLM was Firmicutes (23.21%), whereas, at the other two orchards, Bacteroidetes was the most abundant phylum (23.78% at JC and 25.50% at WJ). The top three phyla comprised 62.82% of the observed taxa in the three orchards. Only 5.95% of observed taxa were not among the top 10 phyla, which were Bacteroidetes, Firmicutes, Proteobacteria, Chloroflexi, Acidobacteria, Actinobacteria, Verrucomicrobia, Cyanobacteria, Thaumarchaeota, and Thermotogae (**Figure 3**).

### 3.4 Comparing of the microbiomes of NF and MF groups

**Figure 4:**
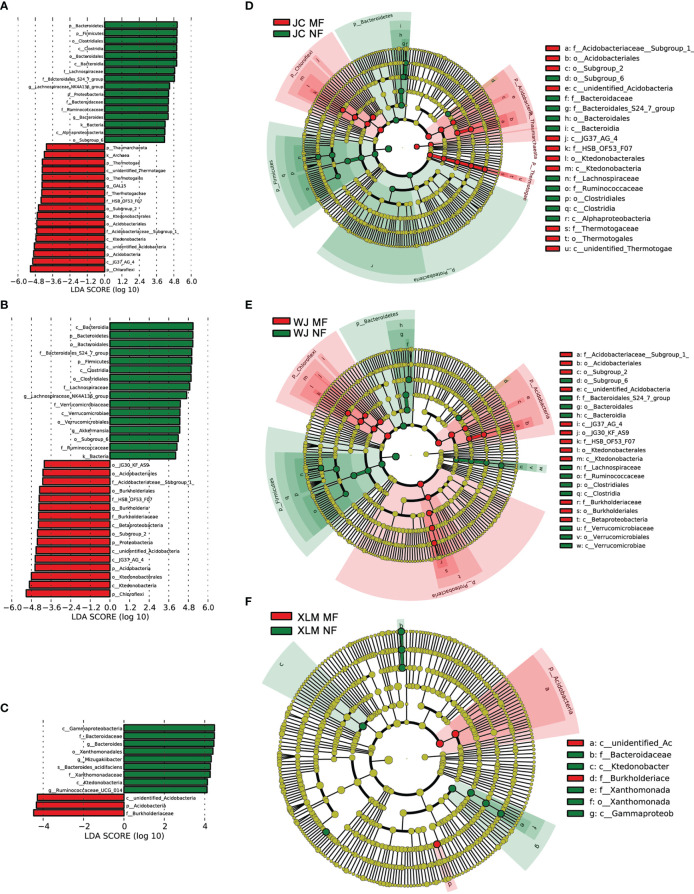
Differences in bacterial taxa abundances in the samples of the MF and NF groups. The forest plots show the linear discriminant analysis score (effect size), indicating the significant differences between the MF and NF groups from **(A)** JC, **(B)** WJ, and **(C)** XLM. The cladograms, which were generated using the linear discriminant analysis of effect size method, indicate the phylogenetic distribution of microbes in the MF and NF groups from **(D)** JC, **(E)** WJ, and **(F)** XLM.

**Figure 5:**
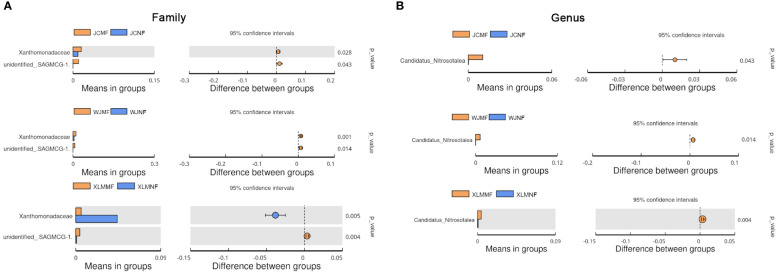
Extended error plots identifying significantly different taxa at the **(A)** family and **(B)** genus levels.

To analyze the differences in the soil microbiome between the NF and MF groups, a linear discriminant analysis of effect size was conducted. The results show that Burkholderiaceae and Acidobacteria were abundant in the MF groups. These two bacterial taxa also showed significantly different abundances (p<0.05, Student’s t-test; **Figure 4**). At the family level, Xanthomonadaceae was significantly more abundant in all the MF groups compared to that in the NF groups (p<0.05). SAGMCG-1 was significantly more abundant in the MF groups from JC and WJ than in the MF group from XLM (p<0.05, **Figure 5A**, **Supplementary Table 3**). At the genus level, *Candidatus Nitrosotalea* was overrepresented in all the MF groups compared to that in the NF groups (p<0.05, **Figure 5B**, **Supplementary Table 4**).

### 3.5 Function of prokaryotic communities in soil from roots of ‘Orah’

**Figure 6:**
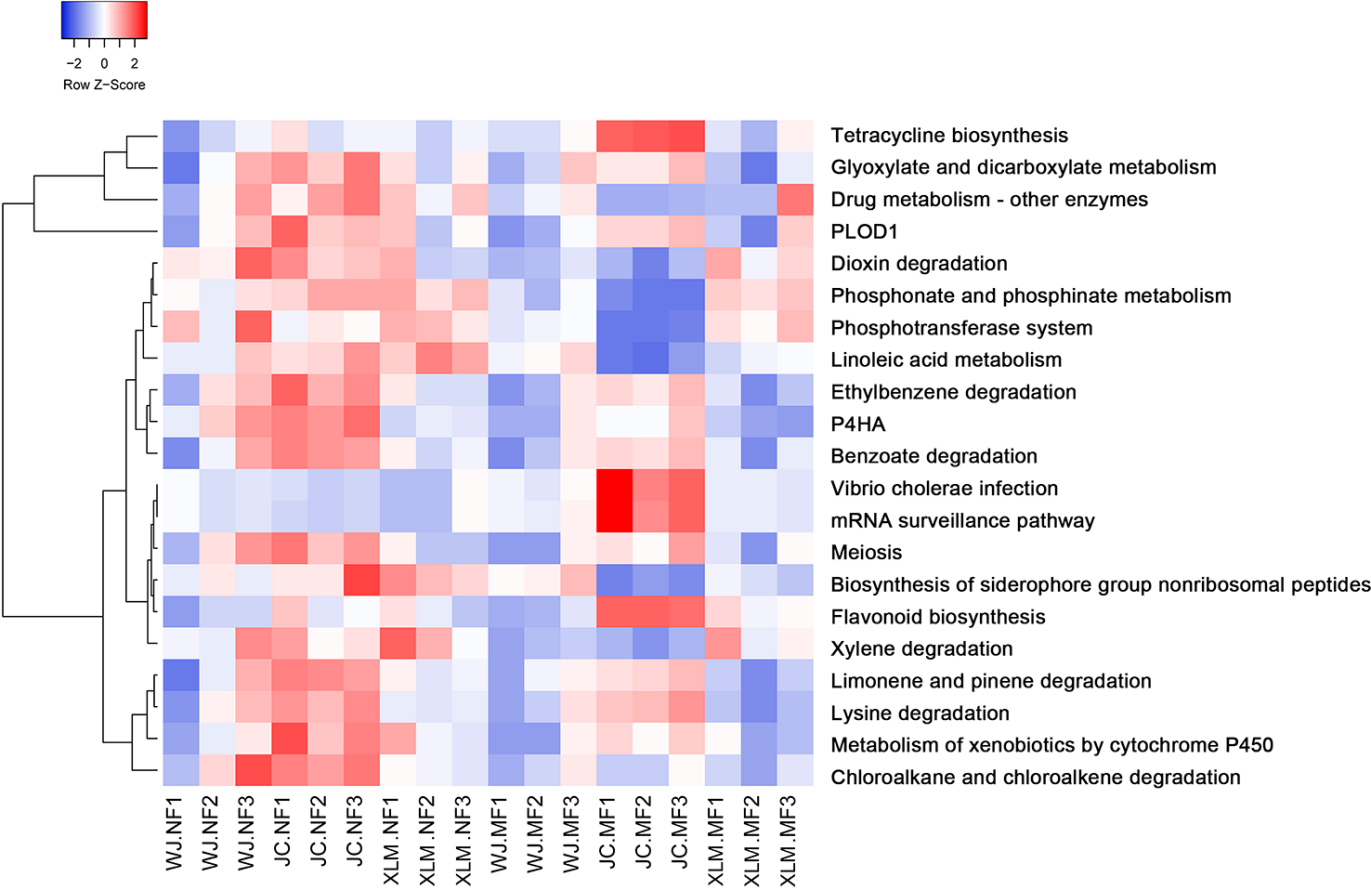
Heatmap of the KEGG analysis of the functional profiles of the prokaryotic communities.

KEGG analysis was performed to predict and investigate the functional profiles of the prokaryotic communities. The results showed that 21 pathways were significantly different between the NF and MF groups (p<0.05, **Figure 6**, **Supplementary Table 5**). Several metabolic pathways were significantly more enriched in the NF groups than those in the MF groups, such as tetracycline biosynthesis, glyoxylate and dicarboxylate metabolism, flavonoid biosynthesis, xylene degradation, limonene and pinene degradation, lysine degradation, metabolism of xenobiotics by cytochrome P450, and chloroalkane and chloroalkene degradation. The KEGG pathways for dioxin degradation, phosphonate and phosphinate metabolism, phosphotransferase system, linoleic acid metabolism, ethylbenzene degradation, prolyl 4-hydroxylase, and benzoate degradation were more enriched in the MF groups than those in the NF groups (**Figure 6**).

## 4 Discussion

In the present study, ‘Orah’ trees with healthy fruit and marbled fruit were sampled to reveal their differences in agronomic traits and soil microbiome in the root regions. To assess potential pathogenic microbes related to marbled fruit disease, PCA and clustering analyses were conducted to determine the diversity of the microbiome. Marbled fruit disease, which occurs due to multiple factors, such as pathogenic microbes, can be relieved by soil-based probiotic treatments (Vuong et al., 2017). Finding and utilizing beneficial microorganisms to improve the quality of citrus fruit is a worthwhile research task. Three different orchards were included in the present study to eliminate the effects of varied natural growth conditions. Moreover, the cultivation and management techniques were similar among these orchards, which approximated those of commercial production. However, under these conditions, 5–10% plants still developed this disease and knowledge on its occurrence from a microbiome view is limited. Therefore, this study investigated the differences in agronomic variables and soil microbiomes from the root regions of NF and MF groups of ‘Orah’.

Many investors who bear the risks brought by MF disease desire to solve this issue. A previous study showed that approximately 57.14% of plants with MF also suffered from yellow vein disease and 100% were infected with the *Citrus tristeza* virus (Bettini et al., 2018). Furthermore, several diseases in citrus that occur due to microbial or viral infections affect fruit production. In America, a stubborn disease of citrus caused by *Spiroplasma citri* results in smaller, deformed fruits with lower yields (Mello et al., 2010). Citrus yellow vein clearing virus causes yellow vein disease in lemons, which leads to yellowing, bright veins, and leaf drop, resulting in a decrease in yield (Liu et al., 2020a). This virus can be spread by grafting, tools, seedlings, and scions (Zhen et al., 2015). Additionally, *Aphid leguminosae* and *Aphid spiraea* can spread this disease in lemons (Tannice and Eric, 2013; García et al., 2016). Some lemon trees are cut down once MF occurs. To date, specific treatments for MF disease are lacking. Furthermore, the pathogenesis of MF may result from a virus or other microbes. The common treatment strategies, such as hormonal treatment and additional fertilization, cannot cure the disease (Banyal and Sharma, 2015; García et al., 2016). Hence, developing new probiotics is important for this disease (Luang-In et al., 2020). Hence, the present study compared the agronomic characteristics of the leaves and fruits of plants with and without MF disease. The disease did not affect the leaf histology, but only lead to low fruit yields. PCA indicated that the difference in microbiomes was higher among the orchards than that between the NF and MF samples. Therefore, these results imply that MF disease affects fewer aspects than previously considered. Although the Shannon and Simpson indices were higher in the MF groups than those in the NF groups at WJ and XLM, no significant difference in the diversity of the soil microbiomes of NF and MF groups was found at JC. Previous studies showed that diversity indicators, such as the Shannon and Simpson indices, were affected by plant biostimulant treatment for endemic huanglongbing (also known as citrus greening disease) (Castellano-Hinojosa et al., 2021) and reared host plants (Meng et al., 2022). Surprisingly, the present results showed no significant differences in diversity between the soil from the roots of the NF and MF groups. We propose that the different conditions such as temperature, water and location among the orchards affected the results.

The taxonomic analysis of healthy ‘Orah’ microbiomes showed that the predominant taxa in three orchards were Bacteroidetes, Firmicutes, and Proteobacteria; this is similar to previous studies. For example, in a study regarding the microbial profiles of affected intensive citrus orchards in Shuitianba town (31°4′ N, 110°41′ E), Zigui City, Hubei, China, the five predominant phyla were Acidobacteria, Bacteroidetes, Firmicutes, Gemmatimonadetes, and Actinobacteria (He et al., 2022). This similarity shows that the compositions of the microbiome are similar among various citrus orchards, and these predominant phyla dominate the microbial community. The microbiome analysis of NF and MF groups of ‘Orah’ suggested that Burkholderiaceae and Acidobacteria were the most abundant in MF groups. Burkholderiaceae includes several plant pathogens, including *Rhizobium* spp. and *Agrobacterium* spp. (Krimi et al., 2002; Deakin and Broughton, 2009). Moreover, at the family and genus levels, Xanthomonadaceae and *Candidatus Nitrosotalea*, respectively, were significantly more abundant in the MF groups and those in the NF groups. Within the Xanthomonadaceae family, genera such as *Xanthomonas* and *Stenotrophmonas* contain several species that are plant pathogens (Ryan et al., 2009). Members of the genus *Candidatus Nitrosotalea*, which includes *Candidatus Nitrosotalea devanaterra* and *Candidatus Nitrosotalea* sp. Nd2, have been found in acidic soils as ammonia-oxidizing archaea (Lehtovirta-Morley Laura et al., 2016). *Candidatus Nitrosotalea* spp. were also found in soil microbial community structures in the soil of rice-frog cultivation and high-quality grassland topsoils (Yi et al., 2019). The functions of these microbes are associated with nitrogen cycle. Nevertheless, the mechanism causing the high concentrations of these microbes in the soil around MF plants remains understudied. We propose that *Candidatus Nitrosotalea* has a novel function in MF that requires further investigation. Additionally, the present study found functional differences in several metabolic pathways in the prokaryotic soil communities between the NF and MF groups. The enriched pathways in the NF groups, including flavonoid biosynthesis and metabolism of xenobiotics by cytochrome P450, contributed to fruit yields, whereas the enriched pathways in the MF groups, such as linoleic acid metabolism, ethylbenzene degradation, prolyl 4-hydroxylase, and benzoate degradation were a possible reason for the low weight and quality of the fruit.

## 5 Conclusion

The agronomic characteristics and soil microbiomes from the root areas were compared between NF and MF groups of ‘Orah’ trees. The plants showed no significant differences between groups; however, the fruits of the MF group were lower quality and lighter weight than those of the NF group. The microbiomes showed no significant differences between the two groups, which was inferred from the alpha and beta diversity analyses. The taxonomy of the microbiomes showed that Burkholderiaceae and Acidobacteria were predominant in the MF groups. At the family and genus levels, Xanthomonadaceae and *Candidatus Nitrosotalea*, respectively, were significantly more abundant in the MF groups than those in the NF groups. The functional analysis by KEGG pathways suggested that the most abundant differing pathways between both groups were those related to metabolism. Thus, these findings provide valuable information regarding the control of MF disease.

## Data availability statement

The datasets presented in this study can be found in online repositories. The names of the repository/repositories and accession number(s) can be found in the article/**Supplementary Material**.

## Author contributions

QH designed the study and wrote the manuscript. NW, JL, ZZ, CW, ST, FL, GL and HH performed the experiment and analyzed the data. HL performed the bioinformatic analysis. CH edited the manuscript. DC, SW and ZQ designed supervised the work the work. All authors contributed to the article and approved the submitted version.
