# Supplementary figures and images for: Soil bacterial communities associated with marbled fruit in Citrus reticulata Blanco ‘Orah’
Source: Front Plant Sci. 2023 May 8;14:1098042. doi: 10.3389/fpls.2023.1098042 (PMC10200933; doi:10.3389/fpls.2023.1098042)

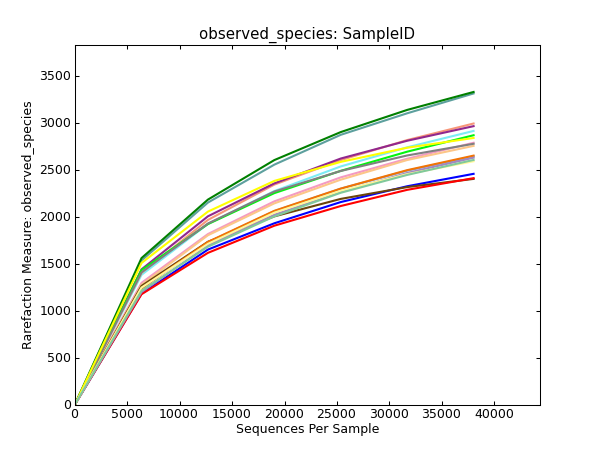

Supplement: Supplementary file 1 [file Image_1.png]
